# Effect of functional electrical stimulation-based mirror therapy using gesture recognition biofeedback on upper extremity function in patients with chronic stroke: A randomized controlled trial

**DOI:** 10.1097/MD.0000000000036546

**Published:** 2023-12-29

**Authors:** Young-Soung Kim, Jun-Young Song, Sam-Ho Park, Myung-Mo Lee

**Affiliations:** a Department of Physical Therapy, Graduate School, Daejeon University, Daejeon, Republic of Korea; b Department of Physical Therapy, Daejeon University, Daejeon, Republic of Korea.

**Keywords:** biofeedback, cerebrovascular disorders, electric stimulation therapy, mirror therapy, physical therapy, rehabilitation, stroke

## Abstract

**Background:**

Mirror therapy (MT) is an intervention used for upper extremity rehabilitation in stroke patients and has been studied in various fields. Recently, effective MT methods have been introduced in combination with neuromuscular electrical stimulation or with electromyography (EMG)-triggered biofeedback. The purpose of this study was to investigate the effects of functional electrical stimulation (FES)-based MT incorporating a motion recognition biofeedback device on upper extremity motor recovery to chronic stroke patients.

**Methods:**

Twenty-six chronic stroke patients with onset of more than 6 months were randomly assigned into experimental group (n = 13) and control group (n = 13). Both groups participated in conventional rehabilitation program, while the control group received conventional MT intervention and the experimental group received FES-based MT with motion recognition biofeedback device. All interventions were conducted for 30 min/d, 5 d/wk, for 4 weeks. Upper limb motor recovery, upper limb function, active-range of motion (ROM), and activities of daily living independence were measured before and after the intervention and compared between the 2 groups.

**Results:**

The Fugl-Meyer assessment (FMA), manual function test (MFT), K-MBI, and active-ROM (excluding deviation) were significantly improved in both groups (*P* < .05). Only the experimental group showed significant improvement in upper extremity recovery, ulnar and radial deviation (*P* < .05). There was a significant difference of change in Brunstrom’s recovery level, FMA, MFT, and active-ROM in the experimental group compared to the control group (*P* < .05).

**Conclusion:**

FES-based MT using gesture recognition biofeedback is an effective intervention method for improving upper extremity motor recovery and function, active-ROM in patients with chronic stroke. This study suggests that incorporating gesture-recognition biofeedback into FES-based MT can provide additional benefits to patients with chronic stroke.

## 1. Introduction

Stroke is a central nervous system disorder caused by sudden ischemia or bleeding of the cerebral blood vessels, leading to a partial loss of brain function and functional impairment.^[1]^ The major symptoms of stroke include muscle weakness, spasticity, coordination disorders, impaired motor function, and sensory impairment, which are the main obstacles to functional motor recovery in stroke patients.^[2]^

Owing to the learned nonuse tendency to prioritize activity on the non-paretic side during tasks using the upper limbs, there is a greater likelihood of gradual loss of function.^[3]^ Stroke-induced upper limb paralysis disrupts various daily activities and decreases functional independence. Therefore, the functional recovery of the upper limbs is considered one of the most critical goals in stroke rehabilitation.^[4]^

Recently, action observation therapy and mirror therapy (MT) have been actively studied for the treatment of upper limb motor function in patients with stroke.^[5]^ MT was first introduced by Ramchandran in 1996 to treat phantom limb pain observed after amputation, using visual illusions and mirror projections.^[6]^ Based on the principles of brain plasticity, MT is a treatment method that promotes upper-limb functional recovery in patients with brain damage by inducing and promoting movement on the paralyzed side.^[6]^

MT involves placing a mirror parallel to the patient’s midline and positioning the non-paralyzed limb on the opposite side of the mirror so that the image of the non-paralyzed limb is reflected in the mirror and appears as if it is the paralyzed limb.^[7]^ Stroke patients who used their non-paralyzed arm reported increased activation in the affected hemisphere of their brain when they perceived themselves as using their paralyzed arm. This therapy provides positive visual feedback by simulating normal movements.^[8]^ The use of mirrors allows patients to immediately observe the results of their training and enables continuous and repetitive training, making the treatment suitable for stroke patients with hemiplegia.^[9]^

In the field of IT, not only exercise for health promotion but also technology and services for rehabilitation purposes are being developed.^[10]^ Biofeedback based on movement recognition provides real-time audio and visual information on muscle contraction or movement, which promotes the induction of appropriate muscle contractions, proper body alignment, and the production of normal movements. It trains patients to regulate their degree of muscle tension by receiving audio and visual feedback from their electromyographic activity.^[11]^ In addition, functional electrical stimulation (FES) therapy is widely used in rehabilitation training to promote muscle activation by inducing muscle contraction signals in paralyzed muscles, which prevents disuse atrophy and parallel functional movement training. Recent studies have shown that electromyography (EMG)-triggered biofeedback with electrical stimulation is effective in stroke rehabilitation.^[12,13]^ Furthermore, combining FES therapy with EMG-triggered biofeedback and MT maximizes the effects of rehabilitation by providing real-time visual and tactile feedback.^[14]^ However, previous EMG-triggered biofeedback studies have had difficulty in inducing bilateral movement because they rely on signals from the affected side’s EMG signals. In contrast, this study uses a wireless EMG-based motion recognition device to detect movements on the normal side in real time and trigger the same movements on the affected side. This makes it possible to induce bilateral movement.

This study emphasizes the need for diverse research using MT for upper extremity function and motor recovery in patients with stroke. Therefore, this study aimed to investigate the effects of FES-based MT using movement recognition biofeedback devices on upper extremity motor recovery, motor function, active-range of motion (ROM), and activities of daily living (ADL) in patients with chronic stroke.

## 2. Material and methods

### 2.1. Participants

This study was conducted from April 2022 to October 2022; patients were recruited who had been diagnosed with stroke and were undergoing rehabilitation therapy at Y Hospital in City C. The inclusion criteria were as follows: having experienced a stroke at least 6 months prior; scoring at least 24 on the Korean version of the Mini-mental state examination (MMSE-K) with no problem understanding the study procedures and methods^[15]^; having no visual or auditory impairments; Brunnstrom stages 1 to 4 of motor recovery^[5]^; and voluntarily agreeing to understand the purpose of the study and participate in the experiment. The exclusion criteria were: severe cognitive impairment and language disorders, having undergone surgery for upper limb injuries or diseases within the last 6 months, and inability to participate due to pain or discomfort during the study. Prior to the intervention, a sufficient explanation of the experimental procedures and methods was provided, and only those who signed the voluntary consent form were allowed to participate in the study. This study was approved by the Bioethics Committee of Daejeon University and registered in the WHO International Clinical Trials Registry Platform (no. KCT0008140).

### 2.2. Study design and procedures

This study employed a pre-post control group design, and G-power version 3.19(Heinrich-Heine University, Düsseldorf, Germany) was used to determine the sample size.^[16]^ Based on the results of Lee et al’s study, a main effect size of d = 1.28 was calculated, with a significance level (β) of 0.05 and power (1–β) of 0.8, indicating a minimum of 11 participants per group. A total of 26 participants (13 per group) were recruited, accounting for a dropout rate of 15%.^[5]^ Participants were randomly assigned to either the experimental group (n = 13) or the control group (n = 13) using a random number generator program.^[17]^ All participants underwent a general rehabilitation program consisting of neurodevelopmental and occupational therapy. In addition, the control group underwent a standard MT program, whereas the experimental group underwent a MT program based on FES using a motion-recognition biofeedback device. Each group’s MT program was conducted for 30 minutes per session, 5 sessions per week, for 4 weeks. To compare and analyze the effects of the intervention method and differences between the groups, an evaluation of upper limb motor recovery, upper limb function, joint ROM, and ADL were conducted before and after the intervention.

### 2.3. Interventions

#### 2.3.1. General mirror therapy.

The general MT program was modified from the MT program proposed by Lee et al and consisted of 9 movements in 12 categories (Table 1).^[5]^ An acrylic mirror (40 cm wide, 30 cm high, and 1 mm thick) was used to construct the MT box. The box was designed to be foldable and easy to assemble, and Velcro was attached to the bottom for easy folding and storage. The angle of the mirror’s surface can also be adjusted. Participants sat on a chair without a backrest and positioned the affected limbs inside the MT box, while their unaffected limbs were positioned in front of the box. The participants were then instructed to mirror the movements of their unaffected limb using their affected limb while imagining that the reflected image in the mirror was their affected limb. The MT program was conducted 5 times per week for 30 minutes per session over a total of 4 weeks.

**Table 1:** Mirror therapy program.

Mirror therapy program
Staring at your hand in the mirror
Providing sensory stimulation
Wrist flexion/Extension
Forearm pronation/Supination
Radial deviation/Ulnar deviation
Clenched fist and flexing the wrist/Opening the palm and extending the wrist
Fist clenched and ulnar deviation/Palm open and radial deviation
Fist clenched and forearm pronation/Open the palm and forearm supination
90 degree elbow flexion while floating from the floor and wrist flexion/extension
90 degree elbow flexion while floating from the floor and forearm pronation/supination
90 degree elbow flexion while floating from the floor and ulnar deviation/radial deviation
Flexing the wrist while squeezing the ball for sensory stimulation

#### 2.3.2. FES-based mirror therapy using gesture recognition biofeedback.

**Figure 1.**
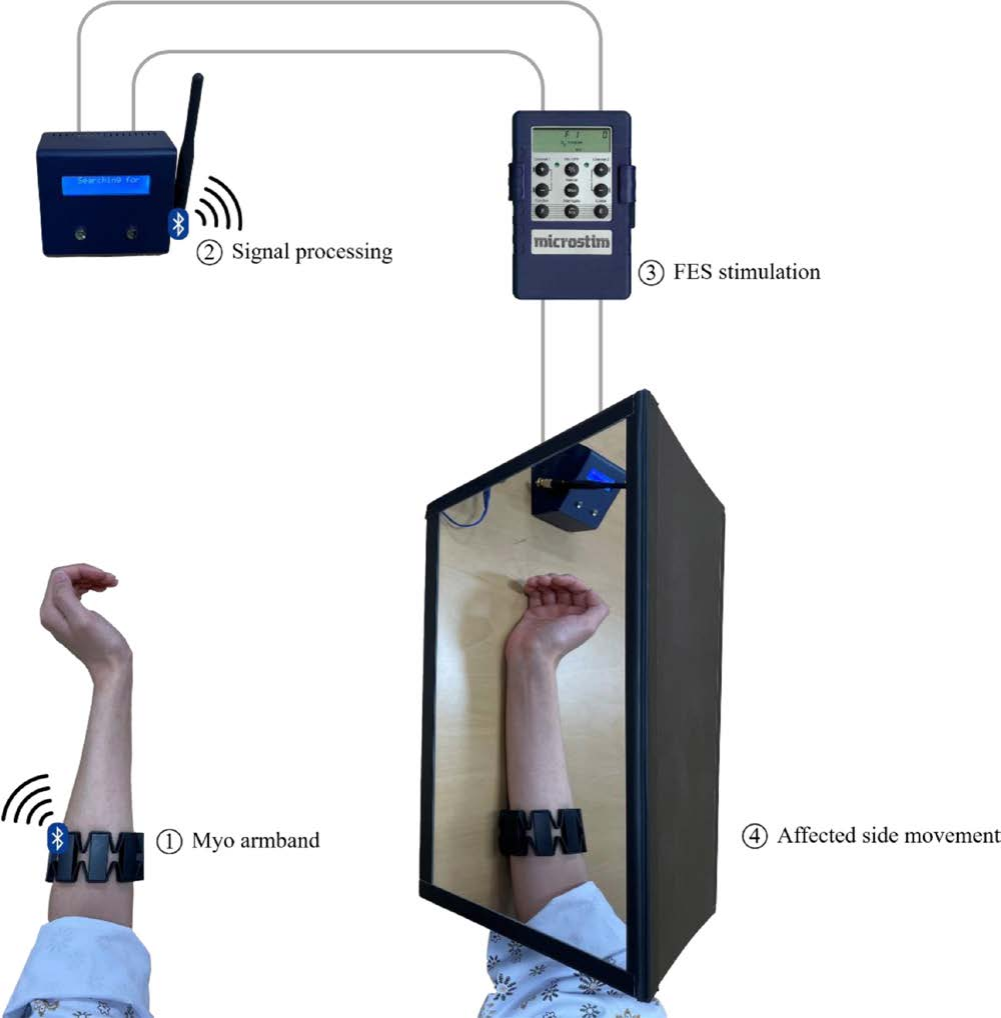
FES-based mirror therapy program.

A MT program based on FES combined with gesture recognition biofeedback was applied to the experimental group, and the following devices were constructed (Fig. 1). The Myo armband (Thalmic Labs, MYO, Waterloo, ON) is an EMG-based gesture-recognition application. It was worn on the participant’s unaffected forearm and detected muscle contraction signals that caused wrist flexion and extension through EMG signals. The detected movements were recognized and transmitted to a receiver via Bluetooth. The receiver converted the signals from the Myo armband into output signals and sent them to the FES device (Sejin Mt, Microstim2, Seoul, Korea) connected to 2 channels. Each channel was attached to a pad placed on the muscles that controlled wrist flexion and extension. For example, when the participant performs wrist dorsiflexion on the unaffected side, the Myo armband recognizes the movement and sends a signal to the receiver, which immediately sends an electrical signal through a specific channel and pad attached to the affected wrist, inducing dorsiflexion movement. The FES pads were attached to the muscles that control wrist flexion and extension on the affected side, and the minimum inducible muscle contraction and the output intensity range that the participant could endure were set. FES mode was set to continue stimulating the affected side as long as the nonaffected side was moving. The ramp-up and ramp-down times were set to 0 s to minimize time delay. After all device settings were completed, the participants in the experimental group were instructed to imagine that the hand reflected in the mirror was their affected hand and to perform MT exercises simultaneously with their unaffected hand.

### 2.4. Assessment methods and procedures

#### 2.4.1. Upper extremity motor recovery assessment.

To evaluate the level of upper limb motor recovery in the participants, a Brunnstrom recovery stage assessment was performed. This assessment tool qualitatively evaluates the recovery process of movement control in the upper limbs, hands, and lower limbs of post-stroke patients and divides the stages of motor recovery into 6 items. The higher the measured score, the higher the level of recovery, and the recovery levels of the upper limbs and hands were evaluated by one physical therapist before and after the intervention. The Rasch reliability of this assessment tool is very high, ranging from 0.91 to 0.92.^[18]^

#### 2.4.2. Upper extremity motor function assessment.

The Fugl-Meyer assessment (FMA) scale was used to evaluate upper limb function. This evaluation tool consists of 6 items: arm and leg function, balance, sensation, joint ROM, and pain, with a total of 226 points using a 3-point scale from 0 (unable to perform) to 2 (complete performance). Higher scores indicated better motor function. In this study, only upper limb function items were evaluated and recorded, consisting of 66 points for the shoulder, elbow, forearm (18 items), wrist (5 items), hand (7 items), and upper limb coordination (3 items). The inter-rater reliability and intra-rater reliability of this evaluation tool were both very high, with *r* = 0.99 and *r* = 0.98, respectively.^[19]^

The manual function test (MFT) was used to evaluate the upper limb function of the participants for comparison. The MFT assesses the ROM and recovery of joint tissues required for the functional movement of the upper limb, with 4 items for arm movement, 2 items for grip strength, and 2 items for finger manipulation. The total score is 32 points, with higher scores indicating better upper limb motor function. The reliability of the evaluation-retest was ICC = 0.994 and the inter-rater reliability was ICC = 0.993, indicating very high reliability.^[20]^

#### 2.4.3. Active ROM assessment.

The joint ROM of the participants was evaluated using a smartphone application angle gauge (Angles Video Goniometer, Nathaniel Cochran, USA). To evaluate the active joint ROM, the flexion/extension, pronation/supination, and ulnar/radial deviation of the forearm were assessed. Andrea et al reported high internal and inter-rater reliabilities with an ICC of.900 when measuring ROM using an application.^[21]^ Joo et al^[22]^ also reported very high reliability, with an ICC of.994 and a validity of.997, when evaluating and reevaluating using an application.

#### 2.4.4. ADL assessment.

The Korean version of the modified Barthel Index (K-MBI) was used to evaluate participants’ ADL. The K-MBI consists of 11 areas: personal hygiene, bathing, feeding, toileting, stair climbing, dressing, bowel control, bladder control, ambulation, and transfer from a chair/bed. Because the transfer from the chair/bed items were evaluated only when walking was difficult, the actual measurement area used in this study was 10. Scores from 25 to 49 indicated severe dependence; 50 to 74, moderate dependence; 75 to 90, mild dependence; and 91 to 100, minimal dependence, with 100 points indicating complete independence for all items. The inter-rater reliability was reported to be between 0.93 to 0.98, and the Cronbach’s alpha was 0.84 by Jung et al^[23]^ Results were collected from the pre- and post-assessments compiled by occupational therapists with more than 5 years of clinical experience.

### 2.5. Statistical analysis

The data collected in this study were analyzed using SPSS software (version 25.0; SPSS Inc., Chicago, IL). Descriptive statistics were used to describe the general characteristics of the study participants, and the normality of the sample was verified using the Kolmogorov-Smirnov test. The before-and-after comparison of the dependent variables according to the intervention method within each group was analyzed using paired *t*-tests, whereas the comparison of dependent variables between groups was analyzed using independent *t* tests and chi-squared (χ^2^) tests. Repeated measure analysis of variance was used to identify the change following time and the effects of interactions between time and group. To compare comparison of dependent variables between groups on the ordinal scale, the Mann–Whitney *U* test was used, and the before-and-after comparison of the dependent variables according to the intervention method within each groups on the ordinal scale, the Wilcoxon signed rank test was used. The statistical significance level (α) for all data was set at less than .05.

## 3. Results

Of the 26 participants who participated in this study, one from the experimental group and one from the control group were excluded due to refusal to participate and early withdrawal, resulting in the final data collection of 12 participants for each group. The results of the normality tests conducted on general physical characteristics such as height and weight indicated that all variables satisfied the assumption of normality (*P* > .05). There were no significant differences in sex, age, height, weight, lesion type, lesion side, or cognition between the 2 groups (*P* > .05). The general characteristics of the participants are shown in Table 2.

**Table 2:** General characteristics.

	Experimental group (n = 12)	Control group (n = 12)	*t*/χ^2^	*P*
Sex (male/female)	9/3	8/4	−0.432	.670
Age (yr)	75.58 ± 4.10	74.91 ± 4.27	0.390	.700
Height (cm)	160.75 ± 6.62	159.25 ± 7.37	0.524	.605
Weight (kg)	55.83 ± 7.46	54.08 ± 9.19	0.512	.614
Lesion type (infarction/hemorrhage)	10/2	11/1	0.596	.557
Paretic side (left/right)	11/1	10/2	0.596	.557
MMSE-K (score)	25.53 ± 0.78	25.25 ± 0.75	0.266	.792

The values are presented mean (SD).

MMSE-K = Mini Mental State Examination-Korean Version.

Brunnstrom scores showed no significant differences between the 2 groups in the pretest. And in experimental group showed significant improvement before and after intervention (*P* < .05), whereas in the control group did not showed significant improvement (*P* > .05) (Tables 3).

**Table 3:** Between group comparison of the Brunnstrom motor recovery stage before and after intervention.

		Experimental group (n = 12)	Control group (n = 12)	Z (*P*)
Brunnstrom-UE (mean rank)	Pre	12.96	12.04	−0.448 (.755)
	Post	15.04	9.96	−0.401 (.078)
	Z (*P*)	−2.460 (.014)*	−1.732 (.083)	
Brunnstrom-hand (mean rank)	Pre	12.00	13.00	−1.909 (0.755)
	Post	14.92	10.08	−1.769 (0.101)
	Z (*P*)	−2.598 (.009)*	−1.414 (.157)	

The values are presented mean rank.

Brunnstrom-UE: Brunnstrom motor recovery stage Upper Extrimity.

Brunnstrom-Hand: Brunnstrom motor recovery stage Hand.

*P < .05.

In FMA and MFT, there was no significant difference between the 2 groups before the intervention. And both groups showed significant improvement before and after the intervention (*P* < .05). Furthermore, there was interaction effect between the group and time in the FMA, and MFTS scores (*P* < .05), with the experimental group showing better results than the control group (*P* < .05) (Table 4).

**Table 4:** Between group comparison of upper extremity motor function assessment before and after intervention.

		Experimental group (n = 12)	Control group (n = 12)	*t (P*)	Time, *F (P*)	Time*Group, *F (P*)
FMA-UE (score)	Pre	24.90 ± 5.47	25.50 ± 4.40	0.270 (.790)	592.007 (.000)*	21.805 (.000)*
	Post	30.80 ± 5.49	29.50 ± 4.58			
	Post-pre	5.90 ± 1.10	4.00 ± 0.67	4.670 (.000)*		
	*t (P*)	16.954 (.000)*	18.974 (.000)*			
MFT (score)	Pre	12.33 ± 1.87	11.50 ± 2.07	1.034 (.312)	662.588 (0.000)*	16.176 (0.001)*
	Post	18.50 ± 2.65	16.00 ± 2.22			
	Post-pre	6.17 ± 1.27	4.50 ± 0.67	−5.000 (.000)*		
	*t (P*)	16.856 (.000)*	23.121 (.000)*			

The values are presented mean (SD).

*P < .05.

FMA-UE = Fugl-Meyer assessment Upper Extrimity.

The A-ROM and K-MBI showed no significant differences between the 2 groups in the pretest, and both groups showed significant improvements in Flexion, Extension, Pronation, Supination, and K-MBI (*P* < .05). Furthermore, there was a interaction effect between the group and time in flexion, extension, pronation, supination, ulnar deviation, and radial deviation, with the experimental group showing better results than the control group (*P* < .05) (Tables 5 and 6).

**Table 5:** Comparison of ROM before and after intervention between groups.

		Experimental group (n = 12)	Control group (n = 12)	*t (P*)		Time, *F (P*)	Time*Group, *F (P*)
Flexion (Angle)	Pre	27.92 ± 11.32	35.50 ± 6.84	−1.986 (.060)	204.193 (.000)*		15.622 (.001)*
	Post	30.42 ± 11.07	36.92 ± 6.65				
	Post-pre	2.5 ± 0.80	1.42 ± 0.51	−3.767 (.003)*			
	*t (P*)	10.856 (.000)*	9.530 (.000)*				
Extension (Angle)	Pre	19.42 ± 9.63	20.00 ± 6.42	−.175 (.863)	24.873 (.000)*		9.344 (.006)*
	Post	23.58 ± 9.22	21.00 ± 6.66				
	Post-pre	4.17 ± 3.49	1.00 ± .85	−2.994 (.012)*			
	*t (P*)	4.141 (.0002)*	4.062 (.002)*				
Pronation (Angle)	Pre	40.42 ± 8.51	44.50 ± 7.86	−1.221 (.235)	24.190 (.000)*		7.275 (.013)*
	Post	44.42 ± 8.81	45.67 ± 7.41				
	Post-pre	4.00 ± 3.46	1.17 ± 1.11	−2.514 (.029)*			
	*t (P*)	4.733 (.002)*	3.626 (.004)*				
Supination (Angle)	Pre	25.58 ± 11.31	25.92 ± 5.92	−0.090 (.929)	26.527 (.000)*		10.843 (.003)*
	Post	29.75 ± 10.91	26.83 ± 5.47				
	Post-pre	4.17 ± 3.30	0.92 ± .90	3.293 (.003)*			
	*t (P*)	4.376 (.001)*	3.527 (.005)*				
Ulnar deviation (Angle)	Pre	16.17 ± 4.13	19.75 ± 5.38	−1.830 (.081)	149.192 (.000)*		7.139 (.014)*
	Post	19.42 ± 4.44	21.83 ± 5.27				
	Post-pre	3.25 ± 1.14	2.08 ± .99	−2.244 (.046)*			
	*t (P*)	9.892 (.000)*	7.244 (.082)				
Radial deviation (Angle)	Pre	12.75 ± 2.30	12.42 ± 2.39	.348 (.731)	37.915 (.000)*		11.702 (.002)*
	Post	15.08 ± 2.39	13.42 ± 2.88				
	Post-pre	2.33 ± 0.78	0.67 ± 1.50	3.421 (.002)*			
	*t (P*)	10.383 (.000)*	1.542 (.151)				

The values are presented mean (SD).

*P < .05.

**Table 6:** Comparison of K-MBI before and after intervention between groups.

		Experimental group (n = 12)	Control group (n = 12)	*t (P*)	Time, *F (P*)	Time*Group, *F (P*)
K-MBI (score)	Pre	43.83 ± 7.93	42.83 ± 7.92	0.599 (.556)	201.611 (.000)	4.115 (.055)
	Post	50.83 ± 7.61	47.42 ± 6.71			
	Post-pre	7.00 ± 2.41	5.25 ± 1.76	−1.307 (.218)		
	*t (P*)	10.053 (.000)*	10.307 (.000)*			

The values are presented mean (SD).

*P < .05.

K-MBI = Korean version of modified Barthel index.

## 4. Discussion

This study investigated the effects of MT based on FES with the addition of a motion-recognition biofeedback device in patients with chronic stroke. The results showed that MT based on FES with the addition of a motion recognition biofeedback device significantly improved upper limb motor recovery, upper limb function, and active joint ROM compared to conventional MT.

Surface EMG can detect weak muscle activation that cannot generate muscle contractions, making it applicable for detecting muscle contraction signals in patients with brain injury who have difficulty inducing movement. Biofeedback training using these signals can aid in the improvement of upper limb function in patients with stroke.^[24]^ A meta-analysis reported that combining biofeedback training with conventional MT resulted in a significant improvement in upper limb function in stroke patients compared to conventional MT alone.^[25]^ In addition, the Myo armband can recognize arm movements without the need for additional electrodes or cables for measuring EMG signals, making it not only an input device, but also an applied rehabilitation device.^[26]^ In a systematic literature review by Marcos-Antón et al, significant improvements in joint ROM, agility, and upper limb function were reported when using a Myo armband for upper limb rehabilitation.^[27]^ In this study, a motion recognition biofeedback device was applied through an 8-channel EMG signal Myo armband for MT based on FES in patients with chronic stroke. Real-time FES was applied to the affected side by assisting 6 movements based on the movement of the unaffected side, indicating its methodological value.

The MT with biofeedback-based FES training conducted in this study and the existing MT share the common treatment goal of inducing activation of the affected side through the use of the unaffected side. However, in this study, a biofeedback-based FES device was used to make actual movement on the contralateral side. The result showed that activation of muscle contraction movements at the extremity such as FES activates neuroplasticity and affects the recovery of motor function, which was significantly different from the conventional mirror therapy group.^[28,29]^

This study examined the effectiveness of MT based on FES with real-time visual and tactile feedback by incorporating a motion recognition biofeedback device in patients with chronic stroke. While Kim and Lee reported significant improvement in elbow and wrist flexion and extension movements despite the use of only 2 channels of electrical signals by performing movements that included elbow and wrist flexion and extension in chronic stroke patients for 6 weeks, this study showed significant improvements in joint ROM through 6 movements using 8 channels of electromyographic signals from the Myo arm band in chronic stroke patients, which raises questions about the previous study’s results.^[30]^ Additionally, although a previous study combined FES with MT, similar to the intervention used in the present study, it was designed using wired connections. While movement on the unaffected side could be transmitted to the affected side, the wired connection might lead to the current flowing back into the unaffected side. In contrast, the Myo arm band device used in this study could prevent interference with EMG signals by wirelessly transmitting the signal and preventing unnecessary electrical stimulation from being transmitted to the unaffected side.

Limitations in daily life are experienced by 70% of patients with stroke along with a decrease in quality of life due to functional impairment of the upper extremities.^[31]^ Therefore, the recovery of upper extremity function is crucial for independent daily living.^[32]^ In this study, we evaluated upper extremity motor recovery using the Brunnstrom recovery stage and found significant differences between the groups. According to Samuelkamaleshkumar et al,^[33]^ significant changes in Brunnstrom recovery stages were observed in patients with acute stroke who underwent MT. In addition, Kim et al reported significant differences in hand and wrist parameters in stroke patients who underwent FES therapy with MT for less than 6 months.^[34]^ Previous studies have shown that intervention in patients with subacute stroke of less than 6 months had an effect on upper extremity motor recovery, unlike in this study. Therefore, the current study has clinical significance in that biofeedback-based FES with MT induced neuroplastic changes through visual feedback and was effective for upper extremity motor recovery, even in patients with chronic stroke lasting more than 6 months.

According to Joo et al,^[35]^ reduced upper limb function in patients with poststroke hemiparesis hinders their independent participation in daily life. In addition, even if the affected limb remains functional, disuse can occur, as the unaffected limb can perform daily activities alone.^[2]^ Therefore, upper limb motor function is considered important. In this study, upper limb motor function was measured using the FMA-UE and MFT, and both the experimental and control groups showed significant differences after the intervention. Paik et al reported significant differences in FMA scores in patients with stroke when MT and electrical stimulation were combined.^[36]^ Jung et al^[37]^ also reported significant differences in both experimental and control groups before and after MT combined with EMG-triggered FES. Kojima et al^[38]^ reported the greatest improvement in wrist items when evaluating the FMA after using the ETMS-MT. The results of this study also support those of previous studies, with significant improvements in upper limb function observed in the item corresponding to the proximal upper limb, indicating that the biofeedback-based FES used in the experimental group could produce more detailed movements with a focus on the forearm.

It has been reported that the range of active joint motion of the upper limb is closely related to the function of the upper limb.^[39]^ In this study, the range of active joint motion of the affected side was measured using an application. The results showed a significant difference between the pre- and post-intervention measurements in the experimental group, and a significant inter-group difference was also observed between. However, there were no significant differences in the ulnar and radial deviations in the control group before and after the intervention. It is suggested that the FES based on biofeedback administered in the experimental group, combined with MT, may have affected the electrical signals of the segmented movements through the myoelectric band, thereby affecting the range of joint motion of the affected side. According to Kojima et al and Saliha et al, significant differences were observed in wrist extension when MT was combined with EMG-based electrical stimulation therapy for 8 and 3 weeks, respectively, in patients with acute and subacute stroke.^[38,40]^ In this study, significant differences were observed in the experimental group’s range of joint motion of the affected side when MT was combined with FES based on biofeedback for the overall movement of the forearm, similar to the results of previous studies.

Despite various interventions, stroke rehabilitation is not sufficient to recover from post-stroke sequelae, and it affects the quality of life of patients with upper limb paralysis.^[40]^ Impaired motor function leads to functional limitations and disability in stroke patients, and recovery of motor function is an important goal in stroke rehabilitation.^[34]^ There was no significant interaction effect between the experimental and control groups on ADLs in this study. In this study, the MBI was used to assess the level of daily living functioning. However, in a study that characterized MBI items, there were items that showed differential item functioning between limb strengths. The relationship between upper extremity motor function and ADLs is influenced by lower extremity motor function,^[32]^ and since the modified modified Badel scale (MBI-K) used to assess ADLs evaluates the overall function of the body, including the lower extremities, not just the upper extremities, it is thought that other functions acted as exogenous variables and affected the results. Nevertheless, both groups showed significant functional improvements in active range of motion and upper extremity motor function from pre- to post-intervention, suggesting that visual feedback through mirror therapy influenced motor function recovery and positively affected activities of daily living.

There were limitations to this study. First, the intervention in this study involved simultaneously performing the same movement on the affected and unaffected sides, which is impractical for daily life applications. Second, the intervention was biased towards upper extremity movements rather than overall upper limb function, which made it difficult to control upper limb proximal muscle function. Third, FES is difficult to apply to a diverse range of movements because of the limited number of channels used. Fourth, the Myo armband has been discontinued and cannot be applied to additional participants. Fifth, the results cannot be generalized to patients with acute or subacute stroke, as this study only targeted patients with chronic stroke. Sixth, the long-term effects of the intervention could not be determined as there were no follow-up observations. Therefore, future studies need to address these limitations by considering the overall upper limb function and daily life activities in patients with chronic stroke and develop more practical interventions.

## 5. Conclusion

This study aimed to investigate the effects of FES-based MT using a movement recognition biofeedback device on the recovery of motor function, ROM, and ADL of the affected upper limb in patients with chronic stroke patients for 4 weeks. Significant improvements were observed in motor function, ROM, and ADL of the affected upper limb in the experimental group. Therefore, this intervention method, which combines FES-based MT with a movement recognition biofeedback device, can encourage active patient participation and is more effective than conventional MT. This method also enhanced the effects of FES.

## Author contributions

**Conceptualization:** Young-Soung Kim, Jun-Young Song, Sam-Ho Park, Myung-Mo Lee.

**Data curation:** Young-Soung Kim, Sam-Ho Park, Myung-Mo Lee.

**Formal analysis:** Sam-Ho Park.

**Methodology:** Young-Soung Kim, Jun-Young Song, Sam-Ho Park.

**Project administration:** Sam-Ho Park.

**Supervision:** Myung-Mo Lee.
